# Performance and Accuracy Comparisons of Classification Methods and Perspective Solutions for UAV-Based Near-Real-Time “Out of the Lab” Data Processing

**DOI:** 10.3390/s22228629

**Published:** 2022-11-09

**Authors:** Zsófia Varga, Fanni Vörös, Márton Pál, Béla Kovács, András Jung, István Elek

**Affiliations:** Institute of Cartography and Geoinformatics, Faculty of Informatics, ELTE Eötvös Loránd University, H-1117 Budapest, Hungary

**Keywords:** UAV multispectral images classifications, decision rules, CTA analysis, PCA analysis, classification’s running time, GIS data NRT processing “out of the lab”

## Abstract

Today, integration into automated systems has become a priority in the development of remote sensing sensors carried on drones. For this purpose, the primary task is to achieve real-time data processing. Increasing sensor resolution, fast data capture and the simultaneous use of multiple sensors is one direction of development. However, this poses challenges on the data processing side due to the increasing amount of data. Our study intends to investigate how the running time and accuracy of commonly used image classification algorithms evolve using Altum Micasense multispectral and thermal acquisition data with GSD = 2 cm spatial resolution. The running times were examined for two PC configurations, with a 4 GB and 8 GB DRAM capacity, respectively, as these parameters are closer to the memory of NRT microcomputers and laptops, which can be applied “out of the lab”. During the accuracy assessment, we compared the accuracy %, the Kappa index value and the area ratio of correct pixels. According to our results, in the case of plant cover, the Spectral Angles Mapper (SAM) method achieved the best accuracy among the validated classification solutions. In contrast, the Minimum Distance (MD) method achieved the best accuracy on water surface. In terms of temporality, the best results were obtained with the individually constructed decision tree classification. Thus, it is worth developing these two directions into real-time data processing solutions.

## 1. Introduction

Today, the enormous development of remote sensing sensors carried on drones [1] has made it possible to record areas with cm spatial resolution. This can help research areas related to precision farming [2,3,4,5,6], such as crop protection and nutrient supply, but can also be involved in Industry 4.0 developments [7,8,9], such as self-driving technology. On the other hand, this research development increasingly requires real-time data processing, allowing their integration into the development of automated “out of the lab” solutions [10,11,12].

However, due to the characteristic of orthorectified, multi-channel images, the size of a single file can easily reach 5–10 GB [13,14,15]. This makes image processing and decision making challenging, especially if we eventually want to perform it all in near-real-time.

For this reason, we primarily wanted to examine image classification algorithms on two configurations closer to the NRT environment, comparing which of them achieves higher accuracy and which can be operated with less running time. In addition to the most used image classification algorithms, two other directions were investigated.

One is the possibility of Principal Component Analysis (PCA) since PCA is a commonly used solution for both field spectroradiometer and hyperspectral data processing due to a large amount of data [16,17,18]. In addition, the results of PCA can be managed more simply in further steps, while the descriptive nature of the differences remains.

On the other hand, there are novel solutions for processing large amounts of data [19,20,21]. Machine learning [22,23,24] or deep learning [25,26,27] analyses are also receiving increasing attention. Therefore, we included them in the study. In addition, object-oriented analysis based on the combination of different features is also a novel approach [28,29,30,31,32], but several other studies [33,34,35,36] have published solutions for so-called classification tree analysis (CTA). These CTA solutions can be easily developed, especially in a Python environment, so in QGIS as well, according to the NumPy ‘where array’ syntax; however, it can also be integrated into the Scikit-learn environment at any time (the Random Forest classification is also based on this principle). That is why we decided to investigate image classification based on individual decision rules, whereby we have developed a rule framework for classification by linking layers representing different measurement units.

Our primary goal was to assess the above algorithms on a centimeter spatial resolution drone image and then evaluate the best runtime algorithms in terms of accuracy. These can help future “out of the lab” developments based on UAV near-real-time data processing.

## 2. Materials and Methods

### 2.1. Study Area

**Figure 1:**
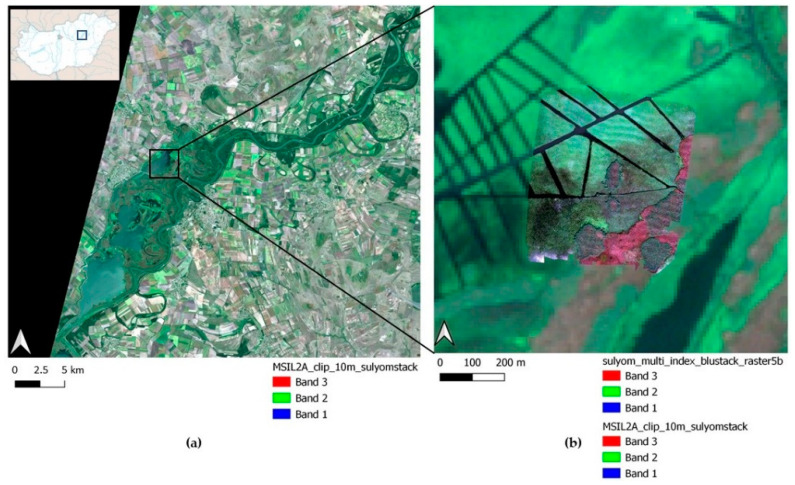
Study area on Lake Tisza, Hungary: (**a**) True color composite of Sentinel MSIL2A satellite image at the same time after preprocessing. (**b**) True color composite of the Micasense Altum 5 band stacked orthophoto after preprocessing (the centroid’s coordinates of the surveyed area: Lat = N47.659, Lon = E20.717, EPSG = 4326).

The field study was conducted on 8 September 2021 with a DJI Matrice 210 v2 RTK drone equipped with a Micasense Altum 6-channel multispectral camera. The location (Figure 1) was a sport fishing area in Lake Tisza, Hungary. The total surveyed area (centroid’s coordinates: Lat = N47.659, Lon = E20.717, EPSG = 4326) was 25.5 ha, which was recorded with three battery replacements (DJI TB55 battery: 7660 mAh/piece; they must be used in pairs for one flight). The flight was performed at an altitude of 70 m, with which the Ground Sampling Distance (GSD) value of the resulting image reached a resolution of 2 cm, which was necessary to meet research purposes.

### 2.2. Processing Workflow and the Examined Algorithms

The acquired UAV images were radiometrically calibrated and orthorectified using Pix4D S.A., Prilly, Switzerland software. To give a sense of the size of this data volume: the raw image file of the flight was 6 times 918 images, of which the pixel count of the single-channel orthophotos produced amounted to more than 612 million pixels. For a 6-channel image, this translates into 6 times 612 million data points for processing. Using the QGIS SCP plugin [37], the spectral values of the sensor bands were adjusted to produce multichannel stacked images in several versions: a 4-band (RGB + NIR, 6 GB in size), a 5-band (RGB + RedEdge + NIR, 8 GB in size) and a 6-band (5band + thermal, 10 GB in size) version.

For all three, we ran the following three most used algorithms for supervised image classification: Minimum Distance (MD), Maximum Likelihood (ML), Spectral Angle Mapper (SAM). The Minimum Distance (MD) classification calculates the Euclidean distance *d*(*x*, *y*) between spectral signatures of image pixels and training spectral signatures, according to the following equation:

(1) $$d\left(x,y\right)=\sqrt{\sum_{i=1}^{n}{\left(x_{i}-y_{i}\right)}^{2}}$$

where *x* is the first spectral signature vector, *y* is the second spectral signature vector and *n* is the number of image bands.

The Maximum Likelihood calculates the probability distributions for the classes, related to Bayes’ theorem (Equation (2)):

(2) $$g_{k}\left(x\right)=\ln p\left(C_{k}\right)-\frac{1}{2}\ln\left|\Sigma k\right|-\frac{1}{2}\left(x-y_{k}\right)t\;\sum_{k}^{-1}\left(x-y_{k}\right)$$

where *C_k_* is the land cover class, *x* is the spectral signature vector of an image pixel, *p*(*C_k_*) is the probability that the correct class is *C_k_*, |Σ*k*| is the determinant of the covariance matrix of the data in class *C_k_*, $\sum_{k}^{-1}$ is the inverse of the covariance matrix and *y_k_* is the spectral signature vector of class *k*.

The SAM algorithms can be written as Equation (3):

(3) $$\theta \left(x,y\right)=cos^{-1}\left(\frac{\sum_{i=1}^{n}x_{i}\;y_{i}}{{\left(\sum_{i=1}^{n}x_{i}^{2}\right)}^{\frac{1}{2}}\times {\left(\sum_{i=1}^{n}y_{i}^{2}\right)}^{\frac{1}{2}}\;}\right)$$

where *x* is the spectral signature vector of an image pixel, *y* is the spectral signature vector of a training area and *n* shows the number of image bands.

Since there was no significant difference in time between the versions of 4 and 5-band (but a drastic increase in time for the 6-band version), we are focusing on the 5-band image results in this paper. On this image we ran the additional machine learning and deep learning algorithms (see later in Table 1), collecting their runtimes.

We examined from machine learning algorithms the Random Forest (RF), the Artificial Neural Network (ANN), and the Support Vector Machine (SVM) algorithm. The Random Forest was based on decision trees and used the Gini index (Equation (4)) as a background of the calculations.

(4) $$Gini\;index=1-\sum_{i=1}^{n}{\left(p_{i}\right)}^{2}$$

where $p_{i}$ is the fraction of items labelled with class *i* in the set. We ran the algorithms with QGIS SCP plugin with ESA SNAP [38] with setting: number of training samples = 5000, number of trees = 10 and with evaluate classifier option. The plugin creates a confidence raster too, where we can see the classification errors, if pixels have low confidence value.

For running ANN classification, we used ORFEO Toolbox [39]. ANN is a nonparametric method of classification with multi-layer perceptron (MLP) using back-propagation [40]. The mathematical background of the process can be seen in Equations (5)–(7).

(5) $$S_{j}=\sum_{i}\;w_{ji}\;p_{i}$$

(6) $$O_{j}=f(S_{j})$$

(7) $$f(S_{j})=\frac{1}{1+e^{-S}}$$

where *p_i_* represents the *i*th input to the *j*th neuron in a specific layer, *w_ij_* represents the weight of the synaptic connection from *i*th input from the previous layer to *j*th neuron in the current layer, *O_j_* is the output from the *j*th neuron in the current layer and *f* represents the transformation function. We ran in ORFEO with the following setting: train method type was back-propagation, the neuron activation function type was symmetrical sigmoid and all other settings were used as default.

The SVM uses a kernel-based method to find a nonlinear projection of the data where the classes are linearly separable. We used the LibSVM method [41,42,43,44], which implements Sequential minimal optimization (SMO) algorithm for kernelized SVM. Along SVM classification, we set the SVM kernel type as polynomial for model type csvc and we checked in the parameters optimization option; all other settings were used as default.

ORFEO-TensorFlow environment was used for the deep learning solution. We built up and ran the CNN model as recommended by Cresser R. in his study [25].

In addition, we ran an unsupervised classification, with clustering ISODATA method, which means an Iterative Self-Organizing Data Analysis Technique [45,46]. It uses spectral distance between image pixels in feature space to classify pixels into a specified number of unique spectral groups. For running this, we used a QGIS-SCP plugin, with settings: distance threshold was 0.01, the number of classes was 6, the max number of iterations was 10, the standard deviation was0.2 and the minimum class size in pixel was 10. We ran this with the ‘seed signature from band values’ option and for the distance algorithm we chose the SAM algorithm. For example, the object-oriented classification is a bit similar to clustering and also begins with large-scale segmentation (in ORFEO).

**Figure 2:**
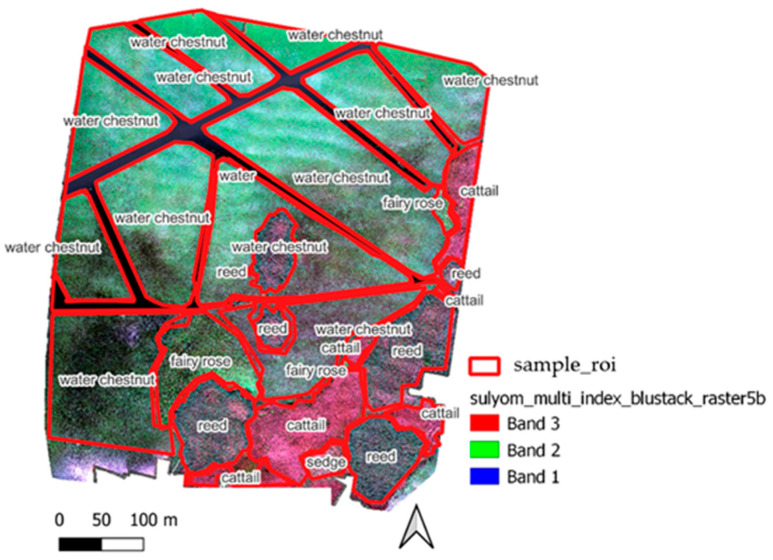
Reference surface vegetation cover based on field survey.

After running the above classifications, we assess the accuracy of the ones with better running time against the reference vegetation cover from the field survey (Figure 2). The classes of vegetation cover were class 1.—water chestnut (*Trapa natans*), class 2.—common reed (*Phragmites australis*), class 3.—cattail (*Typha angustifolia*), class 4.—water surface, class 5.—fairy rose (*Nymphaea alba*) and class 6.—sedge (*Carex acutiformis*). For the supervised classifications, 60% of the field survey vegetation cover was used for the validation training.

As the machine learning and deep learning algorithms took significantly longer time to run and are now less or similar accurate (their Kappa indexes were between 0.55–0.68 for the overall model) than the supervised classifications, their accuracy test results are not detailed in this paper, only their runtime or their estimated memory for full processing.

The results of the three algorithms were compared by an accuracy assessment, examining the overall accuracy in percentage and the Kappa index values. In addition, we examined the spatial evolution of the correct and incorrect pixels using cross-classification.

As the dominant plant in the whole area was water chestnut, which is artificially cut for navigability and water traffic, and there were also larger patches of reeds and cattails, we were also able to use a relatively small area for the training of sedge and fairy rose. In the case of the fairy rose, moreover, it is typical to find it scattered. This is difficult to manage during training, so two larger “patches” were used for fairy rose (class 5) and only one for the sedge (class 6).

In addition to the above, we also examined whether excluding the other two smaller classes from the analysis would increase accuracy for the main four classes.

We have also examined the results of PCA analysis on these images, as in this case we are also working with a larger amount of data due to the spatial resolution. The results of this visual evaluation showed a correlation with the reference data of classes 2, 3, 4, and 5, so we also present an accuracy check of these results.

Due to the previously mentioned characteristics of the fairy rose (class 5), we looked for other methods, examining in which case the predictability of this class can be increased. Thus, a unique decision structure was developed based on the properties of the first 5 classes. During this process, we incorporated into the development of the rule system the thermal properties (after pre-processing, converting the thermal data to Celsius degree), the NDVI features and some single band reflectance data and the specific layers of the PCA analysis. Using different visualizations and band composition layers, we looked for different properties specific to the class of data we were examining, such as the thermal layer, the NDVI layer, the single reflectance layers and the PCA layers. The experience was that if these layers were examined separately with the solutions of the classifications, they did not always achieve sufficient accuracy, whereas if we constructed the rule set from these features individually, combining several features, we achieved better results at the class level. For example, if the Celsius value was within a given range but the NDVI was also within a given range, and we observed some class-specific property in one of the layers of the PCA or the single reflectance layer, we combined them with a Python operator; this provided the basis for the classification. This is similar to the zonal statistical approach, where we can gather statistical data from several raster files in a vector layer, but in our experience the average or minimum/maximum value feature is not always sufficient. Additionally, particular layers may show uniqueness only in some classes and not for all and in different ranges, but if some uniqueness was observed, we linked them together (at least 4–5 unique properties gave a single rule of any class).

We ran the analysis in the Decision Rules menu of the Band calculator module of the QGIS SCP plugin, using Python operators. Then, we assessed its accuracy and whether this solution could be used to increase the proportion of correct pixels for the fairy rose.

**Figure 3:**
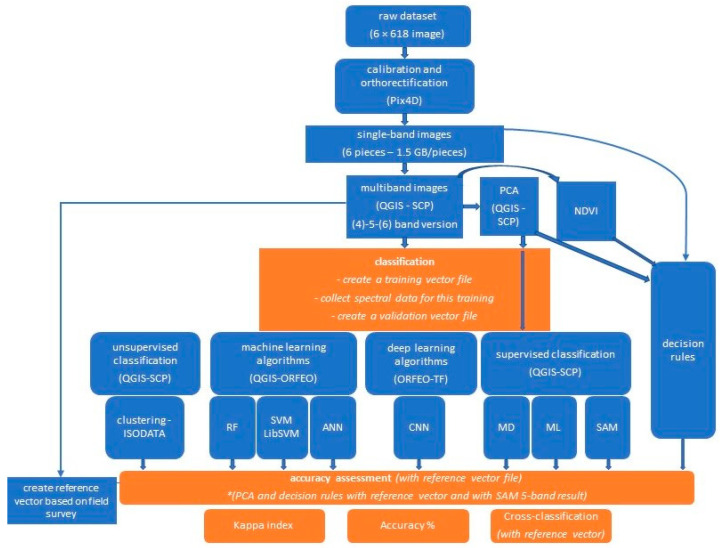
The schematic flowchart of our workflow with the examined algorithms.

The schematic flowchart of the above-detailed workflow is summarized in Figure 3.

All classification runs were performed on two machines in parallel, collecting run-time data. The two used configurations differed mainly in DRAM memory, with 4 GB in one case and 8 GB in the other (the I5 CPU capacity, the Win10 operation system, and the used 3.22 version of the QGIS software were the same). These parameters characterize the memory of the micro- and minicomputer units used by the ‘common’ users and the industrial applications in the widest range (especially in the Central European region). The Raspberry Pi 4 microcomputers are available with 2, 4 or 8 GB of RAM, with industrial units also reaching 4–8 GB or recently starting to exceed this. The requirements of GIS software may differ slightly from this, Python version 3.9 requires a minimum of 4 GB of RAM, QGIS 3.22 or 3.24 recommends 4–8 GB (depending on the used plugins), while ESRI ArcGIS Pro 3.0 at least 8 GB but preferably 16 GB or higher.

Therefore, we wanted to use these two configurations to represent the “out of the lab” computing units.

## 3. Results

### 3.1. Running Time

Table 1 shows the running times by the algorithms used in the study. In each case, we can only see the running time of the given algorithm after the appropriate settings of the same orthorectified, the 5-band stacked image.

The fastest runtime was for ‘Decision rules’, followed by supervised classifications. Although this step requires preparation of the training areas (which will increase the overall runtime, especially for spectral data collection), if we want to automate the same area over time with the same sensor, this step should also be performed only the first time. According to the results, machine learning, object-based or deep learning solutions currently require too much runtime for real-time data processing. To provide a better impression, one of the big advantages of ORFEO is its approach to estimating the total memory required and then dividing the task into parts based on the memory set. For the tested machine learning algorithms, this has now resulted in an average of 23,350 MB of memory for the entire run. In addition, e.g., the first large-scale segmentation step of object-based classification, required 10 h.

### 3.2. Accuracy Assessment Results of the Supervised Classification Algorithms

**Figure 4:**
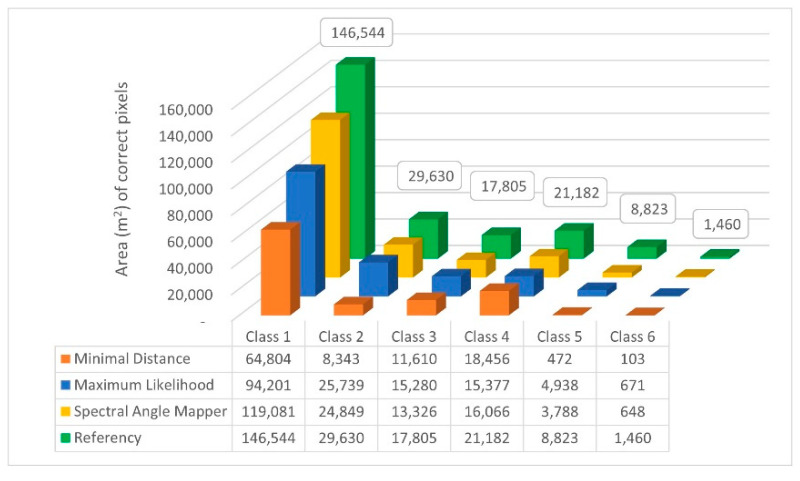
Total area (m^2^) of the correct pixels by classes broken down by the used classification algorithm.

First, we examined the spatial accuracy of the resulting classified images by cross-classification, from which the spatial sum of the correct pixels by classes is shown in Figure 4.

Overall, of the three algorithms, the Spectral Angle Mapper (SAM) algorithm was able to perform the classification with proportionally higher accuracy. For the more dominant vegetation classes (class 1, 2, 3), the SAM and Maximum Likelihood algorithms performed significantly better. In the case of class 1, the difference is explained by changes in the density of the water chestnut, so the increasing number of water pixels leads to an increasing error of the algorithms. On the other hand, it can be observed that, while the Minimum Distance function for the plant cover classes is basically below the accuracy of the other two algorithms, it is characterized by the highest spatial accuracy for the water surface. Therefore, it is recommended to use this algorithm in future analyses of water surfaces.

**Figure 5:**
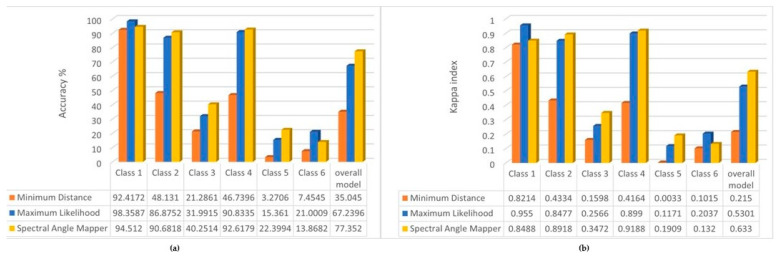
(**a**) Overall accuracy % of the classification algorithms by the classes and the overall model; (**b**) Kappa indexes of the classification algorithms by the classes and the overall model.

The results of the accuracy assessment are shown in Figure 5. This shows how accurately our spectral classes trained during the training process (validation) were able to estimate the correct class for the other pixels (verification), i.e., we compared the classification results with the original reference layer (Figure 2).

It can be seen that for the main classes (1, 2 and 4) our model with Maximum Likelihood and Spectral Angle Mapper behaves with reliable accuracy (Kappa index also shows excellent results over 0.75), while for the classes 3, 5 and 6 the overall accuracy of the model is not satisfactory. This is likely not caused by the size of the study areas, but their spectral values being very similar to classes 1 and 2 (especially for class 2 (reed), which is supposed to cover the inside area with mixed vegetation), which made it difficult to predict accurately. Additionally, in the case of the fairy rose (class 5), the plant characteristics mentioned earlier also make it difficult to train. For the overall model, we can see good Kappa index results produced by the Maximum Likelihood and Spectral Angle Mapper algorithm, which provided the best accuracies.

**Figure 6:**
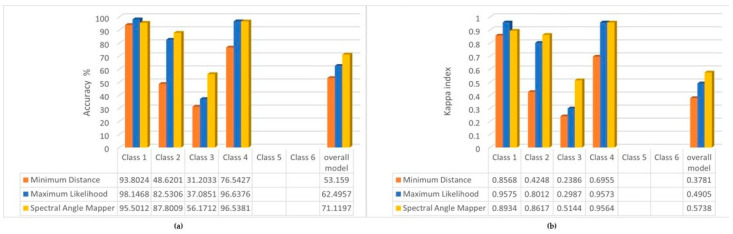
(**a**) Overall accuracy % of the classifications used four classes; (**b**) Kappa index of the classifications used four classes.

For this reason, we investigated whether it would improve accuracy if we removed classes 5 and 6 from the test; the results are shown in Figure 6.

Except for class 2, the accuracy increased for all three other classes. We did not expect an improvement for the entire model, since in this case using the same reference, the missing classes worsen the overall accuracy.

In summary, however, for studies where the analysis is focused on one class, it is preferable to include as few other classes as possible in the training, thus increasing the accuracy of the focused class or parameter. On the other hand, if we analyze vegetation cover, the Spectral Angle Mapper algorithm gives the most accurate results for the multispectral UAV images.

### 3.3. Results of Principal Component Analysis (PCA)

**Figure 7:**
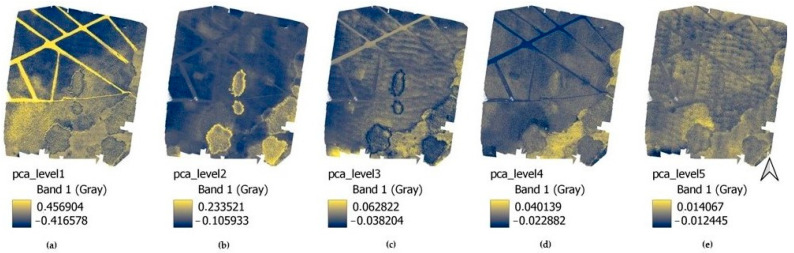
Images of the results of PCA analysis: (**a**) PCA level 1. (**b**) PCA level 2. (**c**) PCA level 3. (**d**) PCA level 4. (**e**) PCA level 5.

The PCA analysis can be used to facilitate the analysis of larger amounts of data. The visual evaluation of the results showed the appearance of the classes investigated in this study (Figure 7).

In (a), the intense yellow color shows the water’s surface (class 4), while dark blue shows the dense cover of the water chestnut (class 1). In addition, it can be used later to help in studies of the density of aquatic vegetation covering the water surface. In (b), the intense yellow corresponds to class 2 (reed); in (c) the dark blue is reed, while the yellow is cattail (class 3). The latter (class 4) is emphasized even more intensely in (d). The (e) is mostly no longer used, although the most intensive yellow (in the middle of the first third of the image) corresponds to the main area of fairy rose.

**Figure 8:**
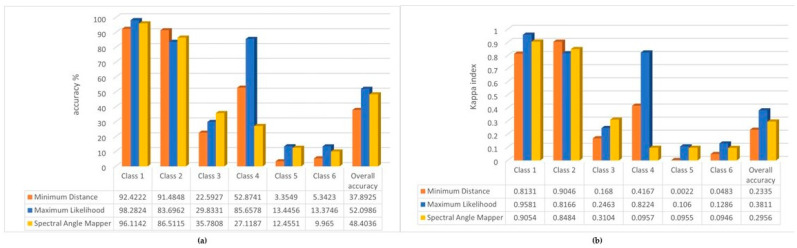
(**a**) The overall accuracy (%) of the PCA analysis and the reference layer. (**b**) The results of Kappa index of the PCA analysis and the reference layer.

**Figure 9:**
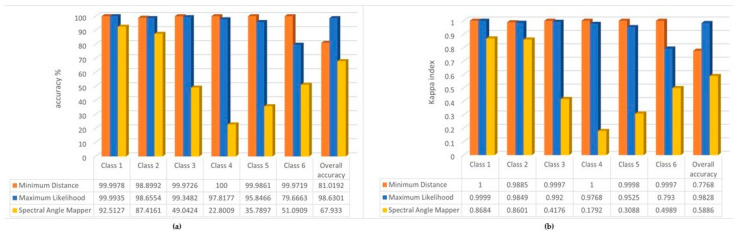
(**a**) Overall accuracy % by the PCA and the UAV ‘original’ classifications. (**b**) Kappa indexes of PCA and the UAV ‘original’ classifications.

Given this experience, we have run the previously tested algorithms for supervised image classification by stacking these layers together and comparing the accuracy analysis with the reference (Figure 8) and with the UAV ‘original’ classification results (Figure 9).

We can see in the results that the accuracy of the Spectral Angle Mapper algorithm has deteriorated significantly, but the Minimum Distance has improved for all classes except class 6 (e.g., from 48% to 91% for class 2). The highest accuracy was achieved by Maximum Likelihood. Compared to the class results of the previous image classification, the accuracy was the same or slightly lower. For the most dominant class 1, the accuracy of both Maximum Likelihood and Spectral Angle Mapper improved minimally. The overall model accuracy decreased.

If we take the original classification results as a reference for the accuracy analysis, we find high accuracy for Minimum Distance and Maximum Likelihood. Additionally, for the overall model accuracy we arrived at the highest values.

### 3.4. Results of the Decision Rules

Besides the fastest runtime, the big advantage of the method is that it allows us to assess many different parameters at the same time. As we increase the number of parameters, we obtain a more accurate prediction solution.

**Figure 10:**
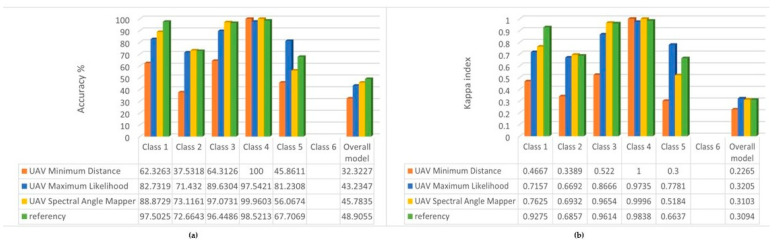
(**a**) Results of accuracy assessment of the decision rules for fairy rose. (**b**) Results of Kappa index of the decision rules for fairy rose.

It is worth paying attention to the focus of the analysis during the runs. The order of the rules can be changed accordingly. For example, if we want to examine the fairy rose more closely, we could improve the results by changing the order of our set of rules for the first five classes. If this rule was examined in 5th place, the system would have selected 863.34 m^2^, while in 3rd place it would have selected 876 m^2^, and if in 1st place it would have selected 915.75 m^2^. The latter was used to perform an accuracy analysis, both against the sorted UAV images and against the reference layer (Figure 10).

If we compare these results with the accuracy of the original supervised image classification with the reference layer (Figure 4), we obtained results that are as good or better for nearly all classes (except for the reed—class 2). This may be because the polygonal-mapped training area assumes that the reed reference is complete, whereas, in reality, it is more likely that the inner parts of the reed reference are covered by mixed vegetation. The decision rule system can likely separate more realistically than the polygon-based supervised classification methods.

Generally, the overall accuracy (in %) and the Kappa index for classes 1, 3 and 4 show good prediction reliability, especially in relation to the reference layer; especially for class 5, while the most accurate result for this class was achieved by the Spectral Angle Mapper algorithm against the reference (overall accuracy 22.4%), the same value has now increased to 67.7%.

For the overall model, however, we do not see the high values that are typical for each class. Derived from our results, this is due to the absence of class 6 and to the fact that, in this case, we obtained the highest number of unclassified pixels. When pixels do not match any of the rules they remain in a separate class, which can be further investigated or used to develop additional rules. For example, in the present study, where the water chestnut (class 1) has become sparse, these pixels are not included in class 1 after the classification. Consequently, in the future, we can use this method to map the density of plant cover more easily. However, the vegetation cover on the water surface (also subtracted now) will also be able to be examined separately and its changes over time tracked.

Another major advantage of this method for real-time image processing is that it can be used to develop a complex set of rules for simple layers (for example, we developed the rules for simple layers with file sizes either below 1 GB (NDVI, thermal band) or between 1–2 GB for single layers (Red, Green, NIR, PCA) as opposed to the 8 GB 5-channel stacked image used in the other analyses. The latter could also explain the drastically lower runtime, while we could see that the accuracy did not necessarily deteriorate.

## 4. Discussion

In image classification, we can find many new approaches that provide good results by incorporating machine learning or deep learning algorithms. At the same time, they require increasing computing capacity, as pointed out by several studies, especially the [25]; which can also draw attention. Therefore, in our research work, we examined the accuracy and the running time of the most important algorithms with configurations that are closer to the NRT environment.

According to our results, although the machine learning and deep learning algorithms achieved adequate accuracy (their Kappa index became 0.55–0.68 for the overall model), their running time was significantly higher; thus, they currently do not allow for their application in the NRT environment. The Spectral Angle Mapper algorithm in supervised classification proved to be the most accurate in this study when working with multispectral data and its running time proved to be significantly better than that of machine learning or deep learning solutions.

However, since PCA analysis (especially after visual evaluation) mapped our most interesting surface cover classes surprisingly well, it is worth further research in UAV image processing. Its running time is slightly longer than the supervised classifications, but it can help in the selection of other complex strategies (e.g., changes in plant cover density) and the resulting, simplified dataset makes other algorithms based on it faster. According to our results, the Minimum Distance and Maximum Likelihood algorithms are recommended for classifications based on PCA results.

In addition, we would like to further investigate Python-based solutions for Decision Rules to create more versatile models (e.g., by mapping changes in the density of vegetation cover on a given surface or by incorporating additional parameters into the model to create a more diverse set of rules). Short runtime, good class-level overall accuracy, and versatile integration into GIS or other systems should be aimed for future near-real-time developments.

With each runtime, we would like to draw attention to the fact that the data processing of UAV images requires further development in the future because these products do not yet allow near-real-time “out of the lab” data processing and automated intervention. In addition, such data processing can only be started after calibration, orthorectification, and other pre-processing solutions, of which orthorectification also takes 12–24 h with similar machine configurations. Of course, it can be accelerated with cloud-based fast data transfer or with larger memory capacity, or with analysis divided into smaller territorial units; however, in terms of runtimes, it requires further development in the future.

The future collection and evaluation of ORFEO data, which will be generated by dividing large amounts of data, could help to define more optimal territorial units. In this case, the log files also record the number of pixels of tasks performed in smaller memory units. Thus, future statistical analyses of these can be used to develop a more optimal size for near-real-time data processing.

One possibility to reduce the running time is either to divide the entire task into subtasks, as ORFEO does, or to simplify the input datasets, as in the case of decision rules (where instead of a multi-channel, 8–10 GB image, containing millions of data points at the same time, we combined simpler data structures).

## 5. Conclusions

In this paper, we highlighted that UAV remote sensing technology, with its fast and increasingly accurate imaging solutions and increasingly versatile sensor systems. These days, this technology allows for more complex data analysis, which enables the development of more versatile forecasting solutions. At the same time, we would like to point out that the increasing number of sensor data may already face data processing difficulties, mainly regarding time, which do not really allow linking them to near-real-time systems (while this is a growing expectation). Thus, more emphasis should be put on the development of this direction or more attention should be devoted to further “out of the lab” research on methods (e.g., PCA, Decision Rules) that, through their simpler data representation, achieve faster runtimes with sufficient accuracy. On the other hand, further research should be conducted towards more optimal data sizes (tiling, sub-tasking, optimal data and image size), which will help near-real-time data processing in the future.

## Figures and Tables

**Table 1:** Running times (in hours) of the represented classification methods.

Classification Method		8 GB DRAM	4 GB DRAM
		Running Times (h)	
unsupervised classification	Clustering (ISODATA)	9.7	>24 h
deep learning	Convolutional Neural Networks (CNN)	>24 h	-
	Object-based classification	>24 h	-
machine learning	Random Forest	7.4	>24 h
	Support Vector Machine (SVM)	10.6	>24 h
	Artificial Neural Networks (ANN)	10.2	>24 h
supervised classification	Minimum Distance	1.2	6.5
	Maximum Likelihood	1.2	6.5
	Spectral Angle Mapper	1.2	6.5
	PCA	3.5	8.5
	Decision rules	0.5	2

## Data Availability

Not applicable.

## References

[1] Fan B., Li Y., Zhang R., Fu Q. (2020). Review on the Technological Development and Application of UAV Systems. Chin. J. Electron..

[2] Tsouros D.C., Bibi S., Sarigiannidis P.G. (2019). A Review on UAV-Based Applications for Precision Agriculture. Information.

[3] Liu J., Xiang J., Jin Y., Liu R., Yan J., Wang L. (2021). Boost Precision Agriculture with Unmanned Aerial Vehicle Remote Sensing and Edge Intelligence: A Survey. Remote Sens..

[4] Jung A., Vohland M., De Marchi M., Diantini A. (2022). Hyperspectral Remote Sensing and Field Spectroscopy: Applications in Agroecology and Organic Farming. Drones Information Technologies in Agroecology and Organic Farming Contributions to Technological Sovereignty.

[5] Willkomm M., Bolten A., Bareth G. (2016). Non-destructive monitoring of rice by hyperspectral in-field spectrometry and UAV-based remote sensing: Case study of field-grown rice in north Rhine-Westphalia, Germany. Int. Arch. Photogramm. Remote Sens. Spat. Inf. Sci..

[6] Gómez-Candón D., de Castro A.I., López-Granados F. (2014). Assessing the accuracy of mosaics from unmanned aerial vehicle (UAV) imagery for precision agriculture purposes in wheat. Precis. Agric..

[7] Hognogi G.-G., Pop A.-M., Marian-Potra A.-C., Someșfălean T. (2021). The Role of UAS–GIS in Digital Era Governance. A Systematic Literature Review. Sustainability.

[8] Chen K., Reichard G., Akanmu A., Xu X. (2021). Geo-registering UAV-captured close-range images to GIS-based spatial model for building façade inspections. Autom. Constr..

[9] Balázsik V., Tóth Z. Az UAV Technológia Pontossági Kérdései. Proceedings of the Conference: Drón Felhasználói Fórum.

[10] Fanta-Jende P., Steininger D., Bruckmüller F., Sulzbachner C. (2020). A versatile UAV near real-time mapping solution for disaster response—Concept, ideas and implementation. Int. Arch. Photogramm. Remote Sens. Spat. Inf. Sci..

[11] Luo F., Jiang C., Yu S., Wang J., Li Y., Ren Y. (2019). Stability of Cloud-Based UAV Systems Supporting Big Data Acquisition and Processing. IEEE Trans. Cloud Comput..

[12] Ampatzidis Y., Partel V., Costa L. (2020). Agroview: Cloud-based application to process, analyze and visualize UAV-collected data for precision agriculture applications utilizing artificial intelligence. Comput. Electron. Agric..

[13] Athanasis N., Themistocleous M., Kalabokidis K., Chatzitheodorou C., Themistocleous M., Rupino da Cunha P. (2019). Big Data Analysis in UAV Surveillance for Wildfire Prevention and Management. Information Systems, Proceedings of the EMCIS 2018, Limassol, Cyprus, 4–5 October 2018.

[14] Huang Y., Chen Z., Yu T., Huang X., Gu X. (2018). Agricultural remote sensing big data: Management and applications. J. Integr. Agric..

[15] Ang K.L.-M., Seng J.K.P. (2021). Big Data and Machine Learning With Hyperspectral Information in Agriculture. IEEE Access.

[16] Agarwal A., El-Ghazawi T., El-Askary H., Le-Moigne J. Efficient Hierarchical-PCA Dimension Reduction for Hyperspectral Imagery. Proceedings of the IEEE International Symposium on Signal Processing and Information Technology.

[17] Licciardi G., Marpu P.R., Chanussot J., Benediktsson J.A. (2012). Linear Versus Nonlinear PCA for the Classification of Hyperspectral Data Based on the Extended Morphological Profiles. IEEE Geosci. Remote Sens. Lett..

[18] Uddin M.P., Mamun M.A., Afjal M.I., Hossain A. (2021). Information-theoretic feature selection with segmentation-based folded principal component analysis (PCA) for hyperspectral image classification. Int. J. Remote Sens..

[19] Wang P., Wang L., Leung H., Zhang G. (2021). Super-Resolution Mapping Based on Spatial-Spectral Correlation for Spectral Imagery. IEEE Trans. Geosci. Remote Sens..

[20] Nasrollahi K., Moeslund T.B. (2014). Super-resolution: A comprehensive survey. Mach. Vis. Appl..

[21] Shang X., Song M., Wang Y., Yu C. (2021). Target-Constrained Interference-Minimized Band Selection for Hyperspectral Target Detection. IEEE Trans. Geosci. Remote Sens..

[22] Inzerillo L., Acuto F., Di Mino G., Uddin M.Z. (2022). Super-Resolution Images Methodology Applied to UAV Datasets to Road Pavement Monitoring. Drones.

[23] Ofli F., Meier P., Imran M., Castillo C., Tuia D., Rey N., Briant J., Millet P., Reinhard F., Parkan M. (2016). Combining Human Computing and Machine Learning to Make Sense of Big (Aerial) Data for Disaster Response. Big Data.

[24] Alexakis D.D., Tapoglou E., Vozinaki A.-E.K., Tsanis I.K. (2019). Integrated Use of Satellite Remote Sensing, Artificial Neural Networks, Field Spectroscopy, and GIS in Estimating Crucial Soil Parameters in Terms of Soil Erosion. Remote Sens..

[25] Cresson R. (2018). A Framework for Remote Sensing Images Processing Using Deep Learning Techniques. IEEE Geosci. Remote Sens. Lett..

[26] Yao X., Yang H., Wu Y., Wu P., Wang B., Zhou X., Wang S. (2019). Land Use Classification of the Deep Convolutional Neural Network Method Reducing the Loss of Spatial Features. Sensors.

[27] Baumgartner S., Bognár G., Lang O., Huemer M. Neural Network Based Data Estimation for Unique Word OFDM. Proceedings of the 2021 55th Asilomar Conference on Signals, Systems, and Computers.

[28] Punitha P.A., Sutha J. (2020). Object based classification of high resolution remote sensing image using HRSVM-CNN classifier. Eur. J. Remote Sens..

[29] Elek I., Cserép M., Kacprzyk J. (2021). Processing Drone Images with the Open Source Giwer Software Package. Proceedings of the Future Technologies, Virtual, 28–29 October 2021.

[30] Mittal P., Sharma A., Singh R., Sangaiah A.K. (2022). On the performance evaluation of object classification models in low altitude aerial data. J. Supercomput..

[31] Śledziowski J., Terefenko P., Giza A., Forczmański P., Łysko A., Maćków W., Stępień G., Tomczak A., Kurylczyk A. (2022). Application of Unmanned Aerial Vehicles and Image Processing Techniques in Monitoring Underwater Coastal Protection Measures. Remote Sens..

[32] Wang H., Duan Y., Shi Y., Kato Y., Ninomiya S., Guo W. (2021). EasyIDP: A Python Package for Intermediate Data Processing in UAV-Based Plant Phenotyping. Remote Sens..

[33] Fekete A., Cserép M. (2021). Tree segmentation and change detection of large urban areas based on airborne LiDAR. Comput. Geosci..

[34] Zawieska D., Markiewicz J., Turek A., Bakuła K., Kowalczyk M., Kurczyński Z., Ostrowski W., Podlasiak P. (2016). Multi-criteria GIS analyses with the use of UAVs for the needs of spatial planning. Int. Arch. Photogramm. Remote Sens. Spat. Inf. Sci..

[35] Bou Kheir R., Bøcher P.K., Greve M.B., Greve M.H. (2010). The application of GIS based decision-tree models for generating the spatial distribution of hydromorphic organic landscapes in relation to digital terrain data. Hydrol. Earth Syst. Sci..

[36] Patel H.H., Prajapa P. (2018). Study and Analysis of Decision Tree Based Classification Algorithms. Int. J. Comput. Appl..

[37] Congedo L. (2021). Semi-Automatic Classification Plugin: A Python tool for the download and processing of remote sensing images in QGIS. J. Open Source Softw..

[38] SNAP—ESA Sentinel Application Platform v8.0.0. https://step.esa.int.

[39] Grizonnet M., Michel J., Poughon V., Inglada J., Savinaud M., Cresson R. (2017). Orfeo ToolBox: Open source processing of remote sensing images. Open Geospat. Data Softw. Stand..

[40] Jensen R.R., Hardin P.J., Yu G. (2009). Artificial neural networks and remote sensing. Geogr. Compass.

[41] Chang C.-C., Lin C.-J. (2011). LIBSVM: A library for support vector machines. ACM Trans. Intell. Syst. Technol..

[42] Haridas N., Sowmya V., Soman K.P. (2015). GURLS vs LIBSVM: Performance Comparison of Kernel Methods for Hyperspectral Image Classification. Indian J. Sci. Technol..

[43] Li Y., Melgani F., He B. Fully Convolutional SVM for Car Detection in UAV Imagery. Proceedings of the IGARSS 2019—2019 IEEE International Geoscience and Remote Sensing Symposium.

[44] Bazi Y., Melgani F. (2018). Convolutional SVM Networks for Object Detection in UAV Imagery. IEEE Trans. Geosci. Remote Sens..

[45] Zheng C., Sun D.-W., Sun D.-W. (2008). 2—Image Segmentation Techniques. Computer Vision Technology for Food Quality Evaluation.

[46] Sirat E.F., Setiawan B.D., Ramdani F. Comparative Analysis of K-Means and Isodata Algorithms for Clustering of Fire Point Data in Sumatra Region. Proceedings of the 4th International Symposium on Geoinformatics (ISyG).

